# The efficiency of risedronate in reducing bone resorption after total hip arthroplasty: a meta-analysis of randomized control trials at a minimum of 6 months’ follow-up

**DOI:** 10.1186/s13018-018-0808-z

**Published:** 2018-04-17

**Authors:** Liqing Yang

**Affiliations:** Department of Orthopedics, Shengjing Hospital of China Medical University, Shenyang, 110004 China

**Keywords:** Risedronate, Total hip arthroplasty, Bone mineral density, Meta-analysis

## Abstract

**Background:**

Recently risedronate is suggested to be effective for the prevention and treatment of for osteoporosis in total hip arthroplasty. This meta-analysis aimes to evaluate the efficacy of risedronate in reducing femoral periprosthetic bone mineral density loss in patients undergoing primary total hip arthroplasty.

**Methods:**

A systematic search was performed in Medline (1966-31 October 2017), PubMed (1966-31 October 2017), Embase (1980-31 October 2017), ScienceDirect (1985-31 October 2017) and the Cochrane Library. Only randomized controlled trial (RCT) were included. Fixed/random effect model was used according to the heterogeneity tested by I2 statistic. Meta-analysis was performed using Stata 11.0 software. The outcome measures included periprosthetic bone mineral density, length of stay and adverse effects.

**Results:**

Four RCTs including 198 patients met the inclusion criteria. The present meta-analysis showed that there were significant differences between treatment groups in terms of periprosthetic bone mineral density in Gruen zones 1 (standard mean difference (SMD) = 0.758, 95% CI 0.469 to 1.047, *P* = 0.000), 2 (SMD = 0.814, 95% CI 0.523 to 1.106, *P* = 0.000), 3 (SMD = 0.340, 95% CI 0.059 to 0.622, *P* = 0.018), 6 (SMD = 2.400, 95% CI 2.029 to 2.771, *P* = 0.000), and 7 (SMD = 2.400, 95% CI 2.029 to 2.771, *P* = 0.000).

**Conclusion:**

Oral risedronate could significantly reduce periprosthetic bone resorption around an uncemented femoral stem (Gruen zones 1, 2, 3, 6, and 7) up to 6 months after THA. In addition, no severe adverse events were identified. Future trials of risedronate treatment after THA should focus on clinically relevant end points such as the risks of fracture and revision arthroplasty.

## Background

Total hip arthroplasty (THA) has become successful surgical procedures for the treatment of end-stage hip osteoarthritis [1]. Cementless THA has been widely accepted to obtain the biological bone fixation of the implant, but proximal bone resorption around the stem occurs frequently with this procedure [2, 3]. The implantation of femoral component may lead to osteopenia of the proximal femur due to stress shielding [4, 5]. Periprosthetic bone loss after THA is associated with reduced bone mineral density (BMD) which increases the risk of migration, implant loosening, and periprosthetic fractures [6].

Numerous articles have focused on the periprosthetic bone metabolism after THA [7]. Bisphosphonates are antiresorptive agent which promotes bone mineralization and inhibits the biological effect of osteoclasts [8]. Substantial randomized controlled trials (RCTs) have demonstrated its beneficial effect on preserving periprosthetic bone in cementless THA [9]. Risedronate is a bisphosphonate with potent antiresorptive activity that is used in the treatment of Paget disease of bone and multiple myeloma [10, 11]. Risedronate can also reduce the risk of vertebral fractures and hip fractures in patients with osteoporosis. It could rapidly reduce bone turnover rates in adult patients at high risk of fractures. In addition, risedronate has the potential efficacy in protecting against osteoporotic fractures and improving periprosthetic bone quality [12, 13]. So far, no approved therapy for BMD loss associated with THA has been reached due to the low evidence level of the current articles.

The use of risedronate for preventing periprosthetic bone loss in THA was seldom published. Therefore, there was not a fully evidence for routine use. Thus, we conduct a meta-analysis from RCTs to evaluate the efficacy of risedronate in reducing femoral periprosthetic BMD loss in patients undergoing primary THA.

## Methods

This meta-analysis was reported according to the preferred reporting items for systematic reviews and meta-analyses (PRISMA) guidelines. All analyses were based on previous published studies; thus, no ethical approval and patient consent are required.

### Literature search

**Fig. 1:**
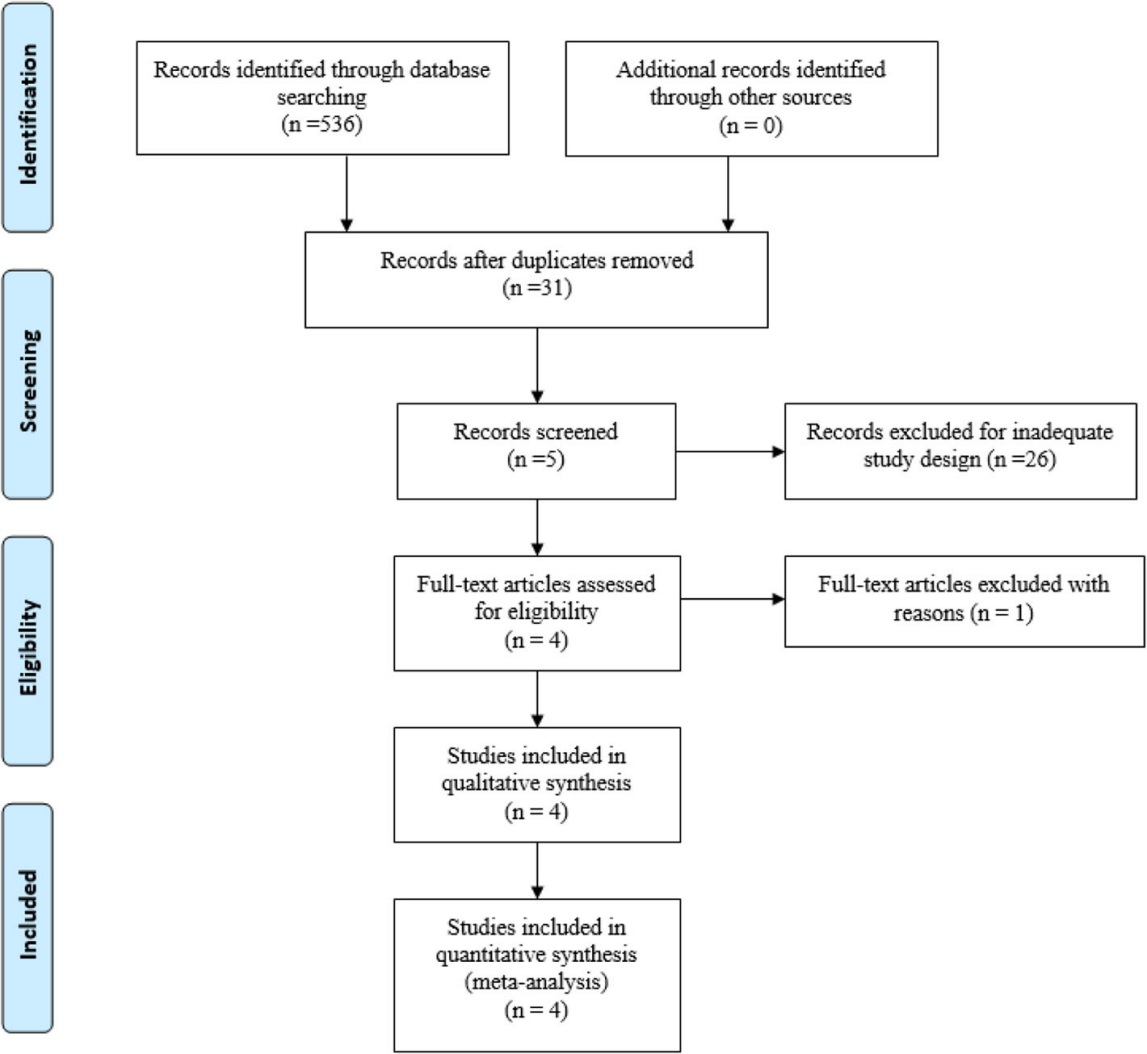
Search results and the selection procedure

Potentially relevant studies were identified from electronic databases including MEDLINE (1966–2017.10), PubMed (1966–2017.10), Embase (1980–2017.10), ScienceDirect (1985–2017.10), and the Cochrane Library. The following keywords were used on combination with Boolean operators AND or OR: “total hip replacement OR arthroplasty,” “risedronate,” and “bone loss.” No restrictions were imposed on language. The bibliographies of retrieved trials and other relevant publications were cross-referenced to identify additional articles. The search process was performed as presented in Fig. 1.

### Inclusion and exclusion criteria

**Fig. 2:**
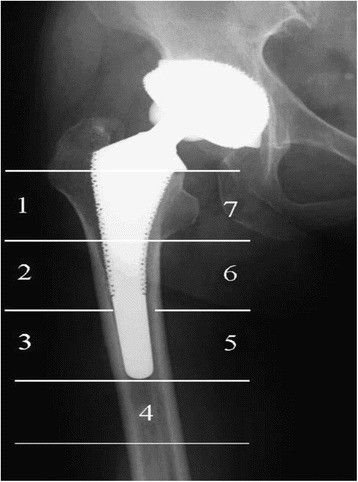
The seven regions of interest based on Gruen zones

The inclusion and exclusion criteria were as follows:

1. Participants: RCTs enrolling adult patients undergoing THA with a diagnosis of end-stage of hip osteoarthritis.
2. Interventions: Experimental groups received oral risedronate.
3. Comparisons: Control groups received equivalent placebo or no treatment.
4. Outcomes: Change in BMD in Gruen zones (Fig. 2) and the occurrence of adverse events.
5. Study design: RCTs were considered as potential relevant included articles in our study.

### Selection criteria

Two reviewers independently scanned the abstracts of the potential articles identified by the above searches. Subsequently, the full text of the studies that met the inclusion criteria was screened, and a final decision was made. A senior author had the final decision in any case of disagreement regarding which studies to include.

### Date extraction

Two of the authors independently extracted data from the included studies. Corresponding authors were consulted for details if it was found to be incomplete. The following data were extracted and recorded in a spreadsheet: first author names, publication year, sample size, baseline characteristics, intervention procedures, anesthesia method, and outcome parameters. Other relevant data were also extracted from individual studies. Primary outcomes were change in BMD in Gruen zones. Secondary outcomes were length of hospital stay and the occurrence of adverse events.

### Quality assessment

Methodological Index for Non-Randomized Studies (MINORS) scale, which assigns scores ranging from 0 to 24, was used to assess the methodological quality of the included studies in the present meta-analysis which was based on the 12 main items. The quality of the evidence for the main outcomes in present meta-analysis were evaluated using the Recommendations Assessment, Development and Evaluation (GRADE) system including the following items: risk of bias, inconsistency, indirectness, imprecision, and publication bias. The recommendation level of evidence is classified into the following categories: (1) high, which means that further research is unlikely to change confidence in the effect estimate; (2) moderate, which means that further research is likely to significantly change confidence in the effect estimate but may change the estimate; (3) low, which means that further research is likely to significantly change confidence in the effect estimate and to change the estimate; and (4) very low, which means that any effect estimate is uncertain.

### Data analysis and statistical methods

Pooling of data was carried out using Stata 11.0 software (The Cochrane Collaboration, Oxford, UK). Statistical heterogeneity was evaluated based on the value of *P* and *I*^2^ using standard chi-square test. When *I*^2^ > 50%, *P* < 0.1 was considered to be significant heterogeneity; random effect model was used for meta-analysis. Otherwise, fixed effect model was performed. The results of dichotomous outcomes (the occurrence of adverse events) were expressed as risk difference (RD) with 95% confidence intervals (CIs). For continuous various outcomes (change in BMD), mean difference (MD) or standard mean difference (SMD) with a 95% CIs was applied for assessment.

## Results

### Search result

A total of 536 studies were identified through the initial search. By scanning the abstracts, 532 reports that did not meet inclusion criteria were excluded from the current meta-analysis. No gray literature was included. Finally, four RCTs [14–17] published between 2006 and 2015 were included in the present meta-analysis; these studies included 97 patients in the experimental groups and 101 patients in the control groups. The duration follow-up ranged from 6 months to 4 years. All included studies were published in English.

### Study characteristics

The sample sizes ranged from 24 to 73 patients. Only studies that included patients with end-stage hip osteoarthritis were included in the present meta-analysis. In these studies, the experimental groups received oral risedronate and the control groups received equivalent placebo or no treatment. The characteristics of the included studies are reported in Table 1. Statistically similar baseline characteristics were observed between groups.

**Table 1:** Trial characteristics

Studies	Year	Reference type	Cases (risedronate /C)	Mean age (risedronate /C)	Female%	Risedronate group	Control group	Follow-up
Kinov	2005	RCT	12/12	58.3/56	62.5%	35 mg risedronate	No treatment	A minimum of half year
Yamasaki	2006	RCT	19/21	66.8/66.7	90%	2.5 mg/day orally	Placebo	A minimum of half year
Skoldenberg	2011	RCT	36/37	61.2/60.3	59%	35 mg risedronate	Placebo	A minimum of 1 year
Muren	2015	RCT	30/31	62.5/60.8	38%	35 mg risedronate	Placebo	A minimum of 4 years

*RCT* randomized controlled trial, *C* control

### Risk of bias

The Cochrane Handbook for Systematic Review of Interventions was consulted to assess risk of bias of the RCTs. All RCTs provided clear inclusion and exclusion criteria and described their randomization methodology, describing the use of computer-generated randomization. All studies reported allocation concealment by closed envelope or other techniques. Double blinding was reported in two RCTs [16, 17]; however, none of the included studies attempted to blind the assessors. An intention-to-treat analysis was not performed in any of the RCTs; therefore, a potential risk of type II statistical error existed. No bias due to selective outcome reporting was identified in the RCTs. The methodological quality assessment is summarized in Table 2.

**Table 2:** Methodological quality of the randomized controlled trials

Study	Random sequence generation	Allocation concealment	Blinding of participates and personal	Blinding of outcome assessment	Incomplete outcome data	Selective reporting	Other bias
Kinov, 2005	Low risk	Low risk	Unclear risk	Unclear risk	Low risk	Low risk	Unclear
Yamasaki, 2006	Low risk	Low risk	Unclear risk	Unclear risk	Low risk	Low risk	Unclear
Skoldenberg, 2011	Low risk	Low risk	Low risk	Unclear risk	Low risk	Low risk	Unclear
Muren, 2015	Low risk	Low risk	Low risk	Unclear risk	Low risk	Low risk	Unclear

### Evidence level

All outcomes in this meta-analysis were evaluated using the Recommendations Assessment, Development and Evaluation (GRADE) system. The evidence quality for most outcome was high (Table 3) which means further research is very unlikely to change our confidence in the estimate of effect.

**Table 3:** The GRADE evidence quality for main outcome

Quality assessment							No. of patients		Effect		Quality	Importance
No. of studies	Design	Limitations	Inconsistency	Indirectness	Imprecision	Other considerations	Risedronate groups	Control groups	Relative (95% CI)	Absolute		
BMD in Gruen zone 1 at 6 months (follow-up 6 months; better indicated by lower values)												
4	Randomized trials	No serious limitations	No serious inconsistency	No serious indirectness	No serious imprecision	None	97	101	–	SMD = 0.758, 95% CI 0.469 to 1.047	High	Critical
BMD in Gruen zone 2 at 6 months (follow-up 6 months; better indicated by lower values)												
4	Randomized trials	No serious limitations	No serious inconsistency	No serious indirectness	No serious imprecision	None	97	101	–	SMD = 0.814, 95% CI 0.523 to 1.106	High	Critical
BMD in Gruen zone 3 at 6 months (follow-up 6 months; better indicated by lower values)												
4	Randomized trials	No serious limitations	No serious inconsistency	No serious indirectness	No serious imprecision	None	97	101	–	SMD = 0.340, 95% CI 0.059 to 0.622	High	Critical
BMD in Gruen zone 4 at 6 months (follow-up 6 months; better indicated by lower values)												
4	Randomized trials	No serious limitations	No serious inconsistency	No serious indirectness	No serious imprecision	None	97	101	–	SMD = 0.275, 95% CI − 0.007 to 0.556	High	Critical
BMD in Gruen zone 5 at 6 months (follow-up 6 months; better indicated by lower values)												
4	Randomized trials	No serious limitations	Serious inconsistency	No serious indirectness	No serious imprecision	None	97	101	–	SMD = 0.204, 95% CI − 0.076 to 0.448	High	Critical
BMD in Gruen zone 6 at 6 months (follow-up 6 months; better indicated by lower values)												
4	Randomized trials	No serious limitations	No serious inconsistency	No serious indirectness	No serious imprecision	None	97	101	–	SMD = 0.503, 95% CI 0.218 to 0.788	High	Critical
BMD in Gruen zone 7 at 6 months (follow-up 6 months; better indicated by lower values)												
4	Randomized trials	No serious limitations	No serious inconsistency	No serious indirectness	No serious imprecision	None	97	101	–	SMD = 2.400, 95% CI 2.029 to 2.771	High	Critical

### Outcomes for meta-analysis

#### BMD in Gruen zone 1 at 6 months

**Fig. 3:**
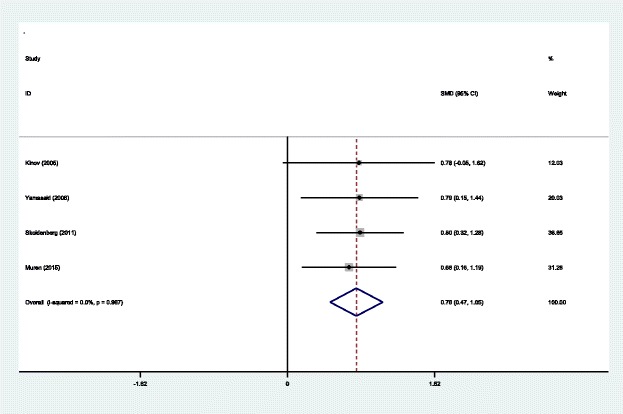
Forest plot diagram showing BMD in Gruen zone 1 at 6 months after THA

Four studies [14–17] reported the outcomes of BMD in Gruen zone 1 at 6 months after THA. A fixed effects model was used because no significant heterogeneity was found among the studies (*χ*^2^ = 0.14, df = 3, *I*^2^ = 0.0%, *P* = 0.987). The pooled results demonstrated that significant difference in BMD in Gruen zone 1 at 6 months was found between the two groups (SMD = 0.758, 95% CI 0.469 to 1.047, *P* = 0.000; Fig. 3).

#### BMD in Gruen zone 2 at 6 months

**Fig. 4:**
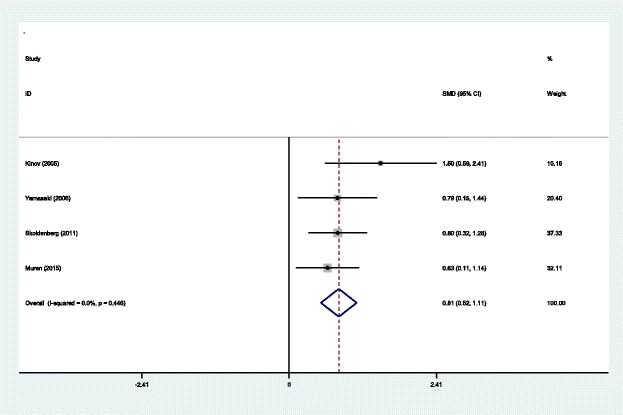
Forest plot diagram showing BMD in Gruen zone 2 at 6 months after THA

Four studies [14–17] reported the outcomes of BMD in Gruen zone 2 at 6 months after THA. A fixed effects model was used because no significant heterogeneity was found among the studies (*χ*^2^ = 2.66, df = 3, *I*^2^ = 0.0%, *P* = 0.446). The pooled results demonstrated that there was significant difference in BMD in Gruen zone 2 at 6 months between the groups (SMD = 0.814, 95% CI 0.523 to 1.106, *P* = 0.000; Fig. 4).

#### BMD in Gruen zone 3 at 6 months

**Fig. 5:**
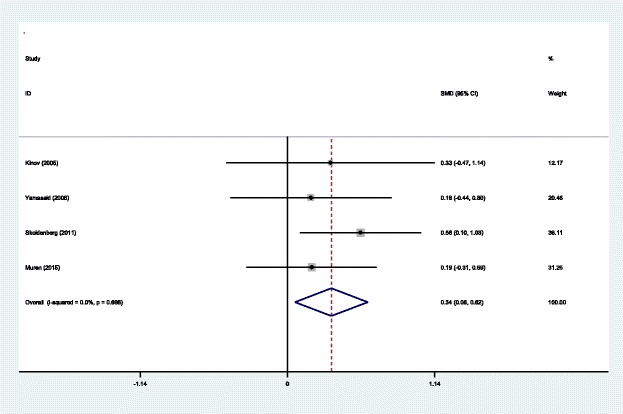
Forest plot diagram showing BMD in Gruen zone 3 at 6 months after THA

Four studies [14–17] reported the outcomes of BMD in Gruen zone 3 at 6 months after THA. A fixed effects model was used because no significant heterogeneity existed among these studies (*χ*^2^ = 1.48, df = 3, *I*^2^ = 0.0%, *P* = 0.686). The pooled results demonstrated that no significant difference in BMD in Gruen zone 3 at 6 months was identified between the groups (SMD = 0.340, 95% CI 0.059 to 0.622, *P* = 0.018; Fig. 5).

#### BMD in Gruen zone 4 at 6 months

**Fig. 6:**
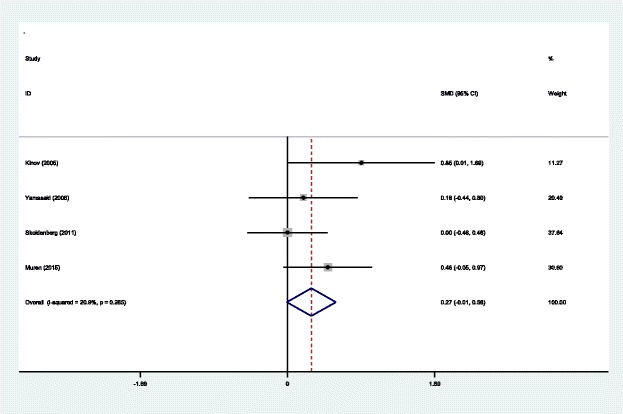
Forest plot diagram showing BMD in Gruen zone 4 at 6 months after THA

BMD in Gruen zone 4 at 6 months after THA was reported in four articles [14–17]. A fixed effects model was applied because no significant heterogeneity was found among these studies (*χ*^2^ = 3.79, df = 3, *I*^2^ = 20.9%, *P* = 0.285). No significant difference was detected in BMD in Gruen zone 4 at 6 months between the two groups (SMD = 0.275, 95% CI − 0.007 to 0.556, *P* = 0.056; Fig. 6).

#### BMD in Gruen zone 5 at 6 months

**Fig. 7:**
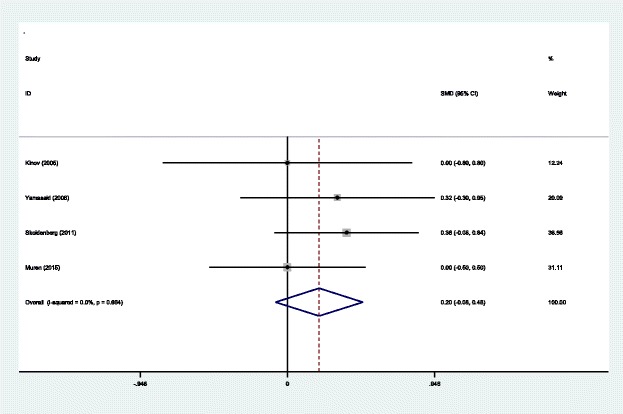
Forest plot diagram showing BMD in Gruen zone 5 at 6 months after THA

BMD in Gruen zone 5 at 6 months after THA was reported in four articles [14–17]. A fixed effects model was used because no significant heterogeneity was found among these studies (*χ*^2^ = 1.58, df = 3, *I*^2^ = 0.0%, *P* = 0.664). The pooled results demonstrated that there was no significant difference BMD in Gruen zone 5 at 6 months between the groups (SMD = 0.204, 95% CI − 0.076 to 0.448, *P* = 0.154; Fig. 7).

#### BMD in Gruen zone 6 at 6 months

**Fig. 8:**
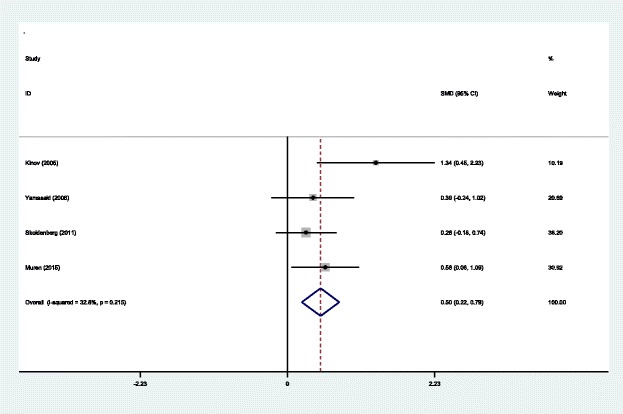
Forest plot diagram showing BMD in Gruen zone 6 at 6 months after THA

Four articles [14–17] reported the outcomes of BMD in Gruen zone 6 at 6 months after THA. A fixed effects model was used because no significant heterogeneity was found among the pooled data (*χ*^2^ = 4.47, df = 3, *I*^2^ = 32.8%, *P* = 0.215). Significant difference in BMD in Gruen zone 6 at 6 months was observed between the two groups (SMD = 0.503, 95% CI 0.218 to 0.788, *P* = 0.001; Fig. 8).

#### BMD in Gruen zone 7 at 6 months

**Fig. 9:**
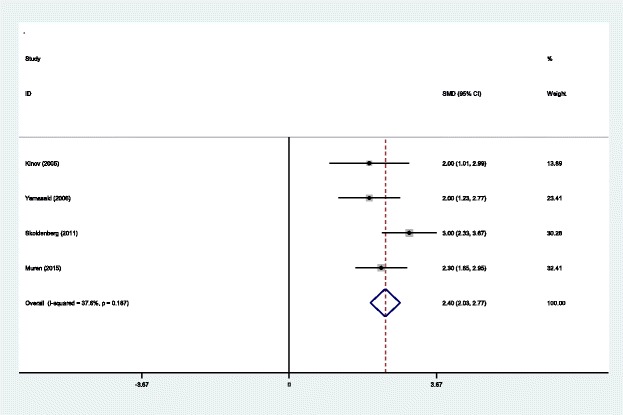
Forest plot diagram showing BMD in Gruen zone 7 at 6 months after THA

Four studies [14–17] reported BMD in Gruen zone 7 at 6 months after THA. A fixed effects model was used because no significant heterogeneity was identified in the pooled results (*χ*^2^ = 4.80, df = 3, *I*^2^ = 37.6%, *P* = 0.187). The pooled results demonstrated that there was significant difference in BMD in Gruen zone 7 at 6 months between the groups (SMD = 2.400, 95% CI 2.029 to 2.771, *P* = 0.000; Fig. 9).

#### Nausea and vomiting

**Fig. 10:**
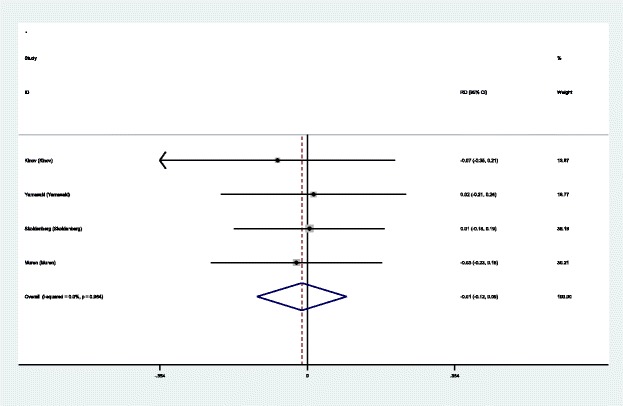
Forest plot diagram showing the incidence of nausea and vomiting after THA

Four studies [14–17] reported the advent events of nausea and vomiting for the groups. A fixed effects model was used because no significant heterogeneity was identified in the pooled results (*χ*^2^ = 0.28, df = 3, *I*^2^ = 0.0%, *P* = 0.964). No significant difference in the nausea and vomiting was found (RD = − 0.013, 95% CI − 0.120 to 0.095, *P* = 0.815; Fig. 10).

#### Hip dislocation

**Fig. 11:**
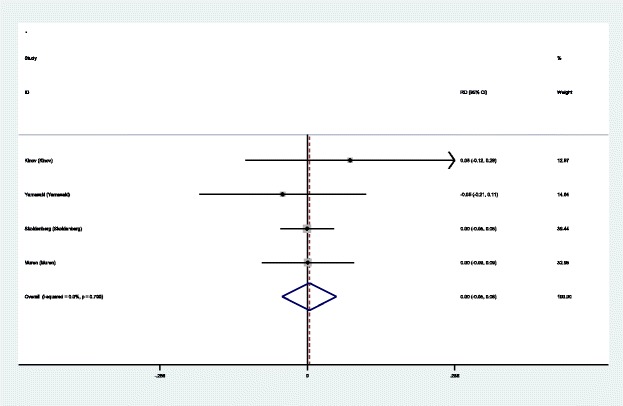
Forest plot diagram showing the incidence of hip dislocation after THA

Four articles [14–17] showed the advent events of hip dislocation for the groups. A fixed effects model was used because no significant heterogeneity was found in the pooled results (*χ*^2^ = 1.01, df = 3, *I*^2^ = 0.0%, *P* = 0.799). No significant difference in the hip dislocation was found (RD = 0.004, 95% CI − 0.049 to 0.057, *P* = 0.876; Fig. 11).

#### Length of hospital stays

**Fig. 12:**
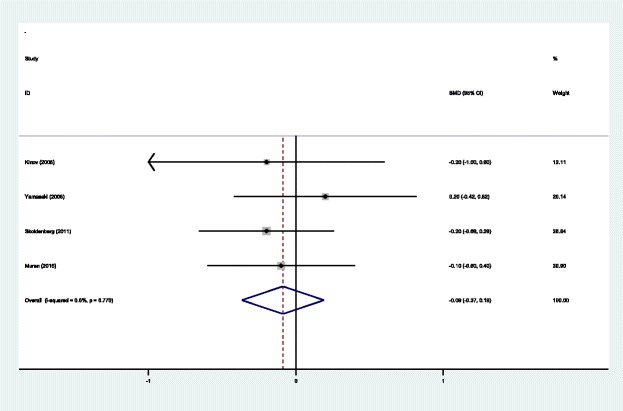
Forest plot diagram showing the length of hospital stay after THA

Four studies [14–17] reported the lengths of the hospital stays for the groups. A fixed effects model was used because no significant heterogeneity was identified in the pooled results (*χ*^2^ = 1.13, df = 3, *I*^2^ = 0.0%, *P* = 0.770). No significant difference in the length of hospital stays was observed between the two groups (SMD = − 0.089, 95% CI: − 0.368 to 0.191, *P* = 0.534; Fig. 12).

## Discussion

To the best of our knowledge, this study is the first meta-analysis to assess the efficiency and safety of oral risedronate in reducing postoperative bone resorption after THA in prospective RCTs. Four RCTs including 198 patients met the inclusion criteria. Three studies applied 35 mg risedronate in treatment groups, and one study took 2.5 mg/day orally. All RCTs were at a minimum of 6 months’ follow-up; therefore, 6-month cutoff was used for results. The periprosthetic BMD in seven regions of interest based on the zones of Gruen which was measured with dual-energy X-ray. Other outcomes were risedronate-related adverse effects including gastrointestinal events and hip dislocation. The most important finding of the meta-analysis was that the oral risedronate was effective in reducing periprosthetic bone resorption in zones 1, 2, 3, 6, and 7 around an uncemented femoral stem up to 6 months after THA compared to the controls. In addition, no increased risk of the incidence of nausea, vomiting, or hip dislocation was identified. All main outcomes in this meta-analysis were evaluated using the GRADE system. The overall evidence quality for each outcome was high, which means that further research is unlikely to change confidence in the effect estimate.

With the aging population, the occurrence of hip osteoarthritis is increasing, and THA is a popular treatment to improve motor function and relieve pain. However, THA was usually associated with proximal bone resorption due to the stress shielding [18]. Several articles have demonstrated that the use of bisphosphonate therapy was effective in reducing stress shielding, and positive effects have been noted in the short- and mid-term time frame [19]. Bisphosphonates hav0065 been reported to decrease wear-induced osteolysis in animal models, and in humans, bisphosphonates can decrease migration of prosthetic implants [20, 21]. The latter is important since early migration of implants is a risk factor for later revision arthroplasty.

Maximal bone remodeling after cementless THA has been reported in the first 6 months after surgery. It was crucial for patients to maintain high level of BMD to decrease the rate of failure of THA (loosening and/or fracture). Eriksen et al. [22] reported the timing of the bisphosphonate administration. Infusions of bisphosphonates in patients with a recent hip fracture led to an increase in total hip BMD as early as 2 weeks. Bhandari et al. [23] showed that bisphosphonates had a beneficial effect in maintaining periprosthetic BMD after total knee arthroplasty (TKA). Despite the published studies, no consensus about effective therapeutic regimen has been reached to maintain periprosthetic BMD after THA due to the small sample size and short-term follow-up.

Risedronate has been proposed to prevent osteoporotic fractures and improve periprosthetic bone quality especially in hip and vertebrae by inhibiting osteoclast activity [24]. Recently, the potential effect of risedronate to prevent or ameliorate periprosthetic bone resorption, osteolysis, and implant migration has been studied. Several articles have reported the short term of outcomes of postoperative risedronate use in preventing periprosthetic bone loss up to a year after THA. In order to standardize the bone density in different area, we applied dual-energy X-ray device to recognize the periprosthetic Gruen zones automatically and compared the BMD after 6 months for patients with or without oral risedronate after THA. The present meta-analysis indicated that oral risedronate led to a significant reduction in bone meta-bolism in Gruen zones 1, 2, 3, 6, and 7 at 6 months after cementless THA. Although no significant difference was found in the Gruen zones 4 or 5, the average level of BMD are higher in intervention groups, which was in accordance to previous studies.

Periprosthetic bone remodeling in the proximal zones is faster than the normal aging of the femoral bone which indicated a potential risk of periprosthetic fracture after THA [25, 26]. Whether there is a distinct relationship between the reduction of BMD around femoral stems and longevity of THA is, however, still very much under debate. Although we have experienced such cases that periprosthetic fractures around femoral components in patients with radiological signs of stress shielding. To link this to scientific evidence of reduction in periprosthetic BMD leading to later fractures or loosening is difficult. Further investigation with large sample size and long-term follow-up are needed.

Duration of oral risedronate after cementless THA remains controversial. Previous studies reported that maximum bone resorption was found in the first 6 months and BMD seemed to stabilize up to 1 year after THA [27, 28]. Arabmotlagh et al. [29] showed that BMD treated for 6 months with diphosphonates was significantly higher than that for 4 months at 6 and 12 postoperative months. Risedronate was allowed to be administrated for more than 6 months with a maintenance dose after cementless THA. Due to the small number of studies available, no reliable evidence regarding the timing of risedronate use after cementless THA was reached. More high-quality RCTs were required for further investigation.

Previous studies have reported an occasional occurrence of a subtrochanteric femoral fracture in patients with osteoporosis after long-term use of alendronate which raised concerns for the bisphosphonate application [30]. The correlation between such fractures and bisphosphonate use continues to be debated. The most common adverse events reported as drug-related were fever, nausea, and vomiting. In our study, all adverse events were mild to moderate in severity and were managed easily with supportive care. There was no significant difference between groups. Additional follow-up was needed to investigate potential severe adverse events.

Several potential limitations of the present meta-analysis should be noted. (1) Only four RCTs were included, and the sample size was relatively small. (2) Some important outcome parameters such as Harris hip scores were not fully described and could not be included in the meta-analysis. (3) Methodological weakness in RCTs should be considered when analyzing the results. (4) Short-term follow-up may lead to the underestimation of the efficiency and safety of risedronate. (5) We assessed for publication bias, due to non-reporting of negative studies, by contacting the principal investigators of unpublished trials registered as completed on trial registries. As there were fewer than 10 studies included, we did not explore publication bias by means of a funnel plot. Further instigation were still necessary.

Despite the aforementioned limitations, this study is the first meta-analysis to assess the efficiency and safety of oral risedronate in reducing postoperative bone resorption after THA in prospective RCTs. More high-quality RCTs with large sample size and long-term follow-up are still required.

## Conclusion

Oral risedronate could significantly reduce periprosthetic bone resorption around an uncemented femoral stem (Gruen zones 1, 2, 3, 6, and 7) up to 6 months after THA. In addition, no severe adverse events were identified. Future trials of risedronate treatment after THA should focus on clinically relevant end points such as the risks of fracture and revision arthroplasty.

## Abbreviations

BMD: Bone mineral density
RCT: Randomized controlled trials
THA: Total hip arthroplasty
